# Alpha-mangostin induces apoptosis through activation of reactive oxygen species and ASK1/p38 signaling pathway in cervical cancer cells

**DOI:** 10.18632/oncotarget.17659

**Published:** 2017-05-07

**Authors:** Chien-Hsing Lee, Tsung-Ho Ying, Hui-Ling Chiou, Shu-Ching Hsieh, Shiua-Hua Wen, Ruey-Hwang Chou, Yi-Hsien Hsieh

**Affiliations:** ^1^ School of Chinese Medicine, College of Chinese Medicine, China Medical University, Taichung, Taiwan; ^2^ Division of Pediatric Surgery, Department of Surgery, China Medical University Children's Hospital, Taichung, Taiwan; ^3^ Department of Obstetrics and Gynecology, Chung Shan Medical University Hospital, Taichung, Taiwan; ^4^ Department of Obstetrics and Gynecology, School of Medicine, College of Medicine, Chung Shan Medical University, Taichung, Taiwan; ^5^ School of Medical Laboratory and Biotechnology, Chung Shan Medical University, Taichung, Taiwan; ^6^ Institute of Biochemistry, Microbiology and Immunology, Chung Shan Medical University, Taichung, Taiwan; ^7^ Graduate Institute of Biomedical Sciences and Center for Molecular Medicine, China Medical University, Taichung, Taiwan; ^8^ Department of Biotechnology, Asia University, Taichung, Taiwan; ^9^ Department of Biochemistry, School of Medicine, Chung Shan Medical University, Taichung, Taiwan; ^10^ Clinical Laboratory, Chung Shan Medical University Hospital, Taichung, Taiwan

**Keywords:** α-mangostin, apoptosis, reactive oxygen species, p38MAPK, cervical cancer

## Abstract

Alpha-mangostin, a natural xanthonoid, has been reported to possess the anti-cancer property in various types of human cancer. However, its effects and mechanism of α-mangostin in cervical cancer remain unclear. We found that α-mangostin effectively inhibited cell viability, resulted in loss of mitochondrial membrane potential (MMP), release of cytochrome C, increase of Bax, decrease of Bcl-2, and activation of caspase-9/caspase-3 cascade in cervical cancer cells. Alpha-mangostin elevated the contents of reactive oxygen species (ROS) to activate p38. Disrupting ASK1/p38 signaling pathway by a specific inhibitor of p38, or by the siRNAs against ASK1, MKK3/6, or p38, significantly abolished α-mangostin-induced cell death and apoptotic responses. Moreover, α-mangostin also repressed tumor growth in accordance with increased levels of p-ASK1, p-p38, cleaved-PARP and cleaved-caspase-3 in the tumor mass from the mouse xenograft model of cervical cancer. In the current study, we provided first evidence to demonstrate that dietary antioxidant α-mangostin could inhibit the tumor growth of cervical cancer cells through enhancing ROS amounts to activate ASK1/p38 signaling pathway and damage the integrity of mitochondria and thereby induction of apoptosis in cervical cancer cells.

## INTRODUCTION

Cervical cancer is a significant cause of cancer death in women worldwide [1]. Although the death rates from uterine cancer declined by more than 80% between 1930 and 2012 due to early detection and prevention through widespread use of the Papanicolaou test or Pap smear, the disease remains a serious health threat. The incidence rate of cervical cancer has decreased by 3.0% per year in women older than 50-years; however, the rate remains stable in women younger than 50-years [2]. The critical factors influencing mortality in cervical cancer patients are recurrence and metastasis to other sites, including lymph nodes [3], lungs [4, 5], and liver [6]. Clinical trials have shown that systemic treatments such as chemotherapy, and combination therapy with chemotherapeutic drugs and angiogenesis blockade prolong overall and progression-free survival of cervical cancer patients with recurrence or metastasis [7].

Poisonous Chinese herbal medicines (PCHM) have been used historically in the treatment of different types of cancer. PCHM-derived natural products, including camptothecin derivatives and vinca alkaloids, have been demonstrated to be promising anti-cancer drugs [7]. In Southease Asia, mangosteen (*Garcinia mangostana Linn*) is considered a medicinal plant and has been used to treat skin infections, wounds, dysentery, various urinary disorders, cystitis, gonorrhea, suppuration, and chronic ulcers [8]. Alpha-mangostin (5-hydroxy-2-methyl-1,4-naphthoquinone) is a natural xanthonoid isolated from the mangosteen tree [9]. It has been shown to possess antioxidant [10] and anti-inflammatory [11, 12] properties. Its anti-cancer activity has also been described in previous studies [13–15], including induction of caspase-independent and caspase-dependent apoptosis in colon cancer cell lines [16]. Results of another study showed that α-mangostin inhibits tumor growth in an HT-29 colon cell xenograft model [17]. In an immunocompetent xenograft model of metastatic mammary cancer carrying a p53 mutation,-α-mangostin induced cell-cycle arrest at G1-phase and apoptotic cell death in several breast cancer cell lines, reducing tumor growth and lymph node metastasis [18]. Similar inhibitory effects of α-mangostin on proliferation and metastasis have been demonstrated in prostate cancer [19]. In addition, α-mangostin repressed epithelial-mesenchymal transition (EMT) by down-regulating the PI3K/Akt pathway [20] and inhibited activation of pancreatic stellate cells (PSCs), an EMT-like process, by suppressing production of hypoxia-driven ROS in pancreatic cancer [13]. These data suggest potential applications of α-mangostin to arrest tumor growth and metastasis in various cancers. However, the anti-cancer effects and molecular mechanisms of α-mangostin in cervical cancer remain unclear.

Apoptosis, or programmed cell death, is generally characterized by distinct morphological changes such as cell shrinkage, pyknosis, and energy-dependent biochemical processes, including extrinsic (or death receptor) and intrinsic (or mitochondrial) pathways [21, 22]. The extrinsic signaling pathways are mediated by the interactions of ligands and death receptors, such as FasL/FasR and TNF-α/TNFR1 [23] In contrast, the intrinsic signaling pathways are initiated by non-receptor-mediated stimuli, including radiation, toxins, and hypoxia, and are mediated via mitochondrial events. All of these stimuli result in opening of the mitochondrial permeability transition (MPT) pore, loss of mitochondrial membrane potential (MMP), and release of cytochrome c to activate apoptosome containing Apaf-1, caspase-9, and caspase-3. These apoptotic mitochondrial events are regulated by members of B cell lymphoma 2 (Bcl-2) family proteins, in which Bax and Bak trigger cytochrome c release and apoptosis, whereas Bcl2, Bcl-xL and Bcl-w, inhibit apoptosis [24].

The mitogen-activated protein (MAP) kinase superfamily controls many biological processes in response to different extracellular stimuli. In general, extracellular signal–regulated kinases (ERKs) are involved in survival and mitogenic signaling, while c-Jun N-terminal kinases (JNKs) and stress-activated protein kinases (SAPKs or p38) MAP kinases are preferentially stimulated by environmental stresses, thereby leading to cell death, survival and differentiation [25]. Apoptosis signal–regulating kinase 1 (ASK1) phosphorylates the mitogen-activated protein kinase kinases (MKKs) MKK4/7 and MKK3/6 to activate both p38 and JNK pathways [26]. In addition, ROS-dependent Akt/ASK1/p38 signaling pathway regulates nickel compound-induced apoptosis in human bronchial epithelial BEAS-2B cells [27]. Results of a previous study showed that genistein promotes TNF-related apoptosis-inducing ligand (TRAIL)-induced apoptosis by suppressing the p38 MAPK pathway in human hepatocellular carcinoma Hep3B cells [28]. In addition, our previous study demonstrated that α-mangostin induces mitochondrial dependent apoptosis in human hepatocellular carcinoma SK-Hep-1 cells by inhibiting the p38 MAPK pathway [29]. However, the anti-cancer properties and mechanisms of α-mangostin in cervical cancer remain unclear. In the current study, we aimed to investigate the roles of α-mangostin in cytotoxicity and apoptotic responses, and to clarify its underlying molecular mechanism.

## RESULTS

### α-mangostin induces apoptosis in cervical cancer cells

**Figure 1:**
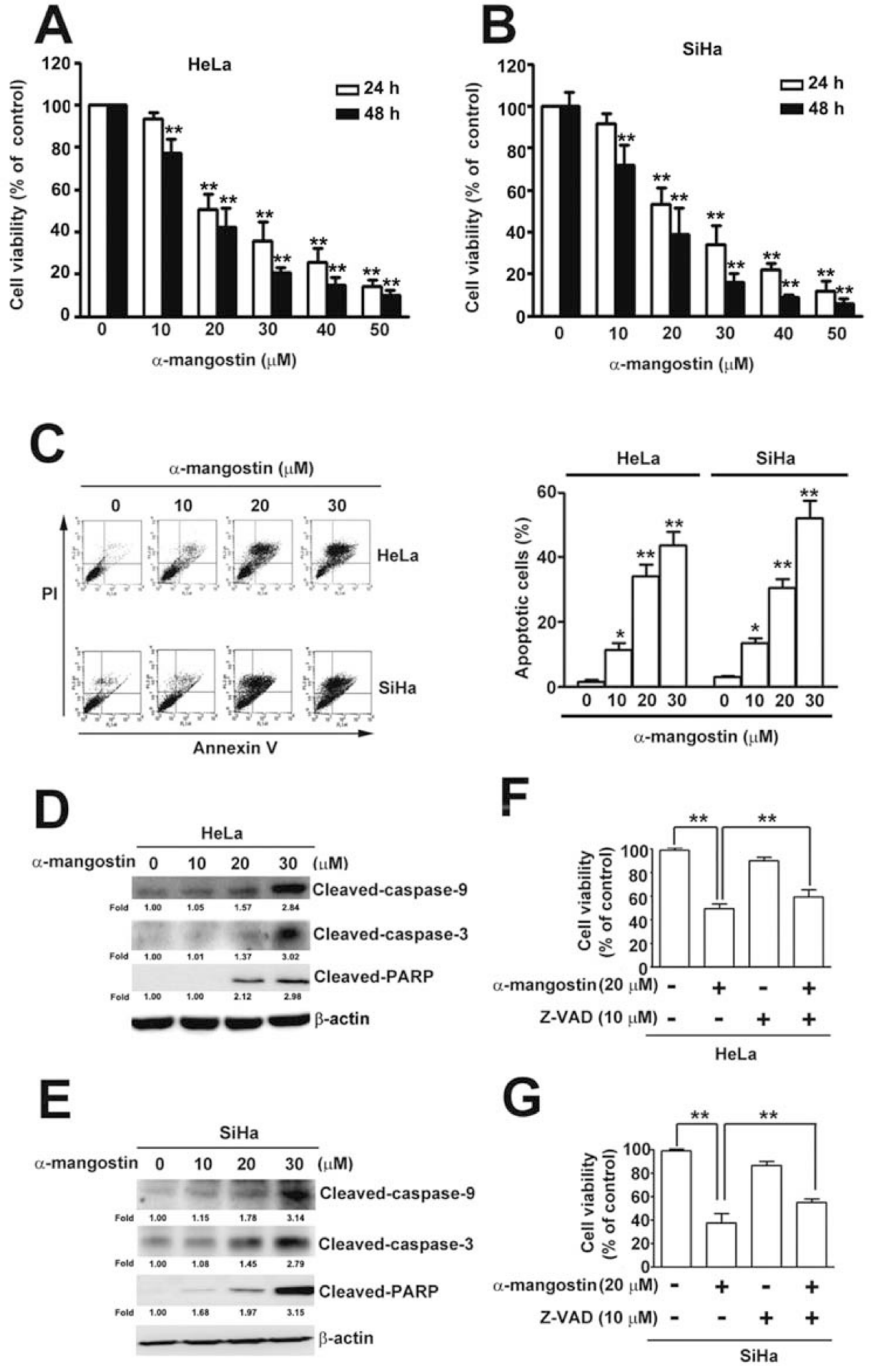
Cytotoxic effects of α-mangostin in cervical cancer cells (**A**) HeLa (**B**) SiHa cervical cancer cells were treated with increased concentrations of α-mangostin (0, 10, 20 and 30 μM) for 24 or 48 h. Cell viability was determined by MTT assay. (**C**) After 24 h of α-mangostin treatment, cells were collected and stained with Annexin V/PI followed by flow cytometryanalysis. Quantitative results of apoptotic cells (Annexin V-stained cells) are shown in the right plot. (**D**, **E**) Cells were treated with indicated concentrations of α-mangostin for 24 h, and then collected and lysed. Expressions of indicated proteins were determined by immunoblotting. β-actin is shown as an internal control. (**F**, **G**) Cells were pre-treated with or without 10 μM of Z-VAD for 2 h followed by incubation with 20 μM α-mangostin for 24 h. Cell viability was evaluated by MTT assay. Data from three independent experiments are represented as mean ± SE. *P < 0.05; **P < 0.01.

To address the cytotoxic effects of α-mangostin in cervical cancer cells, HeLa and SiHa cells were treated with increased concentrations of α-mangostin and the cell viability and apoptotic responses were examined. The results showed that α-mangostin significantly reduced cell viability (Figure 1A and 1B) and induced apoptotic cell death (Figure 1C) in both HeLa and SiHa cells in a dose-dependent manner. In addition to dominant apoptotic cell death, parts of cells underwent necrotic cell death in both HeLa and SiHa cells after treatment of α-mangostin (Figure 1C). Concentration-dependent activation of apoptotic responses, including decreases in pro-caspase-9 and pro-caspase-3, and increases in cleaved-caspase-9, cleaved-caspase-3, and cleaved-PARP were also observed (Figure 1D and 1E). Furthermore, addition of a pan-caspase inhibitor, Z-VAD, significantly reduced α-mangostin-induced cell death in both HeLa (Figure 1F) and SiHa (Figure 1G) cells. These results indicate that caspase-dependent apoptosis is involved in α-mangostin-induced cell death of cervical cancer cells.

### α-mangostin induces loss of mitochondrial membrane potential (MMP) and release of cytochrome C

**Figure 2:**
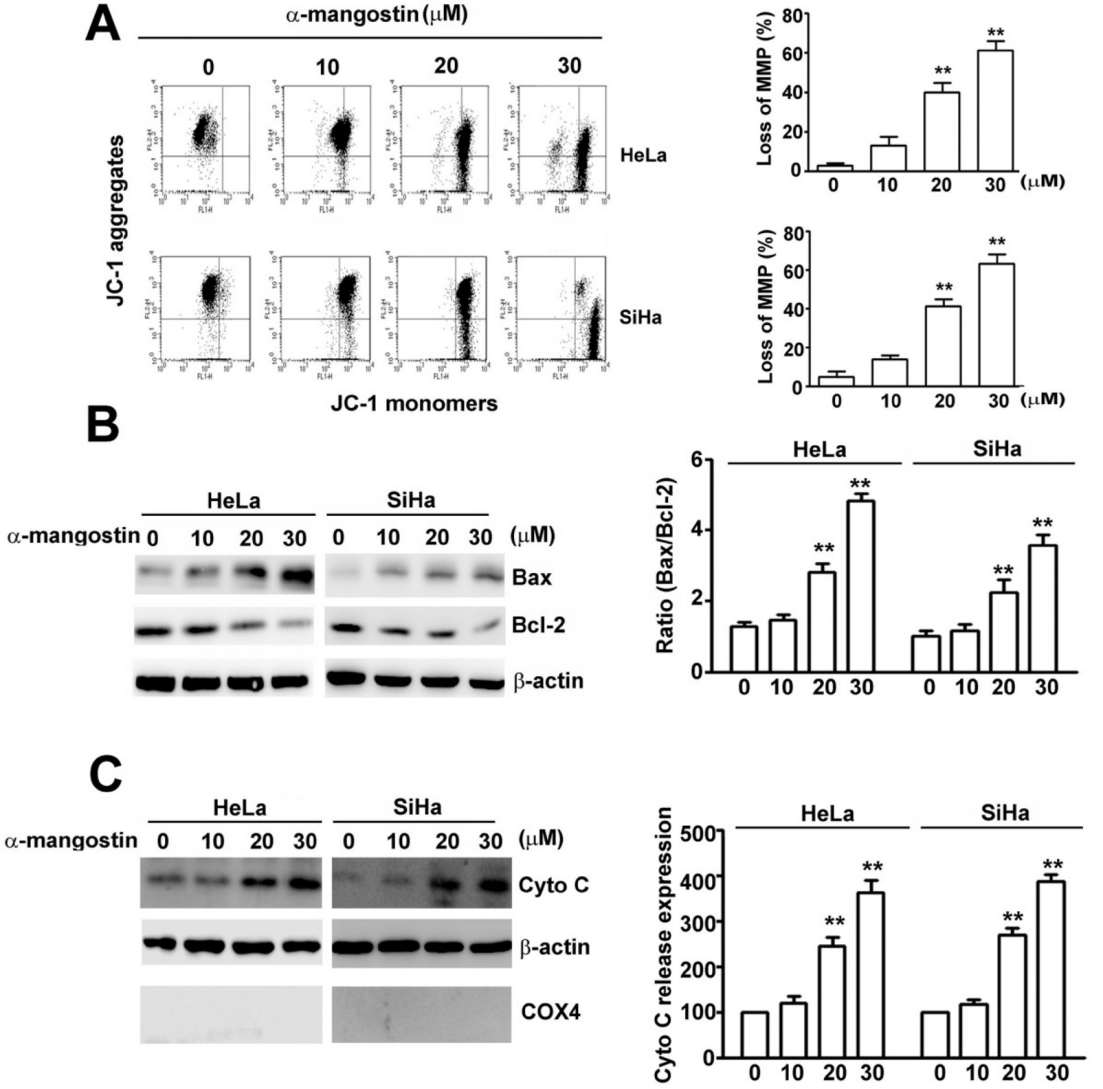
Effects of α-mangostin on apoptotic responses in cervical cancer cells Cells were treated with increased concentrations of α-mangostin (0, 10, 20 and 30 μM) for 24 h. (**A**) The mitochondrial membrane potential (MMP) was determined by JC-1 staining. Damage of mitochondria was evaluated by loss of MMP (a decrease of JC-1 aggregates) as shown in the right plot. (**B**) Cell lysate was collected and expressions of Bax, Bcl-2, and β-actin were examined by immunoblotting. β-actin is shown as an internal control. The ratio of Bax/Bcl-2 in each treatment is shown in the right plot. (**C**) Cytosol and mitochondrial fractions were isolated. Expressions of indicated proteins were determined by immunoblotting. β-actin is shown as an internal control and cytosolic marker. COX4 was used as a mitochondrial marker. Quantitative results of cytochrome C release into cytosol are shown in the right plot. **P < 0.01.

Loss of mitochondrial membrane potential (ΔΨ) is a hallmark for apoptosis, leading to loss of JC-1 aggregates (red fluorescence) and an increase in JC-1 monomers (green fluorescence) [30]. To further demonstrate α-mangostin-induced apoptotic cell death in cervical cancer cells, mitochondrial membrane potential, expression of apoptosis activator, Bax, and anti-apoptotic protein, Bcl-2, and release of cytochrome C were tested. Results revealed that α-mangostin significantly disrupted the integrity of mitochondria measured by loss of MMP in a concentration-dependent manner (Figure 2A). A simultaneous increase of pro-apoptotic proteins, including Bax and cytochrome C, and a decrease in anti-apoptotic protein, Bcl-2, were also observed upon treatment of increased concentrations of α-mangostin in both HeLa and SiHa cells (Figure 2B and 2C). These results α-mangostin induces mitochondrial apoptotic pathway in human cervical cancer cells.

### ROS-activated p38 mediates α-mangostin-induced apoptosis in cervical cancer cells

**Figure 3:**
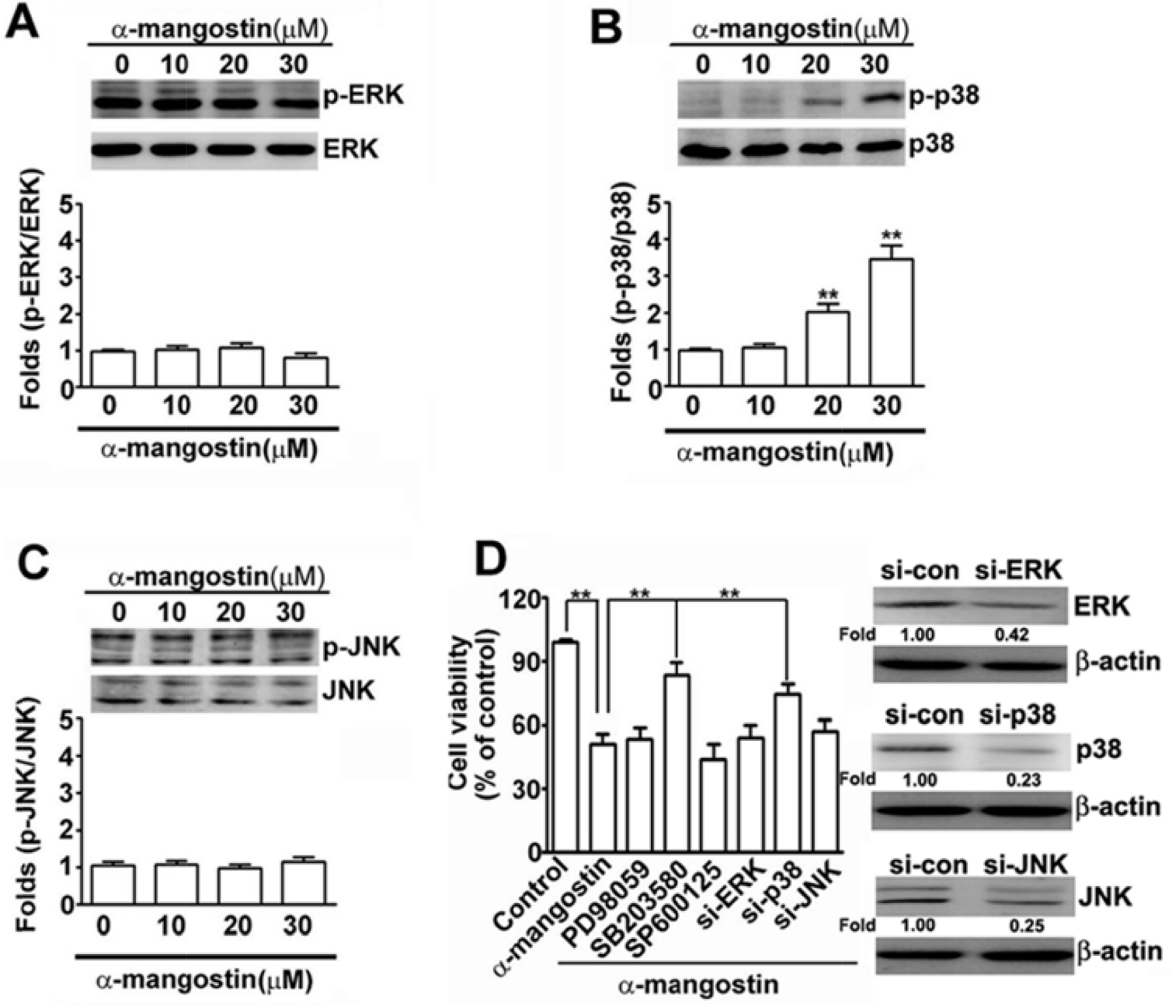
Effects of α-mangostin on MAPK pathways in cervical cancer cells HeLa cells were treated with increased concentrations of α-mangostin (0, 10, 20 and 30 μM) for 24 h. The levels of unphosphorylated and phosphorylated MAPK members, (**A**) ERK, (**B**) p38, and (**C**) JNK, were determined by immunoblotting. Quantitative results are shown in the bottom plot. (**D**) HeLa cells were pretreated with or without 50 μM MAPK inhibitors, PD98059 to ERK, SB203580 to p38, or SP600125 to JNK, for 2 h, and then treated with or without 20 μM α-mangostin for 24 h. Alternatively, HeLa cells were transfected with specific siRNAs against ERK, p38, or JNK for 24 h, and then the transfected cells were treated with 20 μM α-mangostin for 24 h. Cell viability was determined by MTT assay. **P < 0.01.

To address the signaling pathways in α-mangostin-induced apoptotic cell death, several stress-related kinases were examined. While no obvious differences were found in phosphorylation of ERK and JNK (p-ERK and p-JNK), phosphorylated p38 was significantly activated (p-p38) after treatment with 20 μM of α-mangostin in cervical cancer cells (Figure 3A–3C). Moreover, abrogating p38 activity by adding its inhibitor, SB203580, or by transfection of specific siRNA-p38 (si-p38), significantly restored α-mangostin-induced cell death. However, disrupting ERK or JNK activity by PD98059 or SP600125, respectively, or their specific siRNA-ERK (si-ERK) or siRNA-JNK (si-JNK), did not alter α-mangostin-induced cell death (Figure 3D). These results indicate that activation of p38 is involved in α-mangostin-induced cell death in cervical cancer cells.

**Figure 4:**
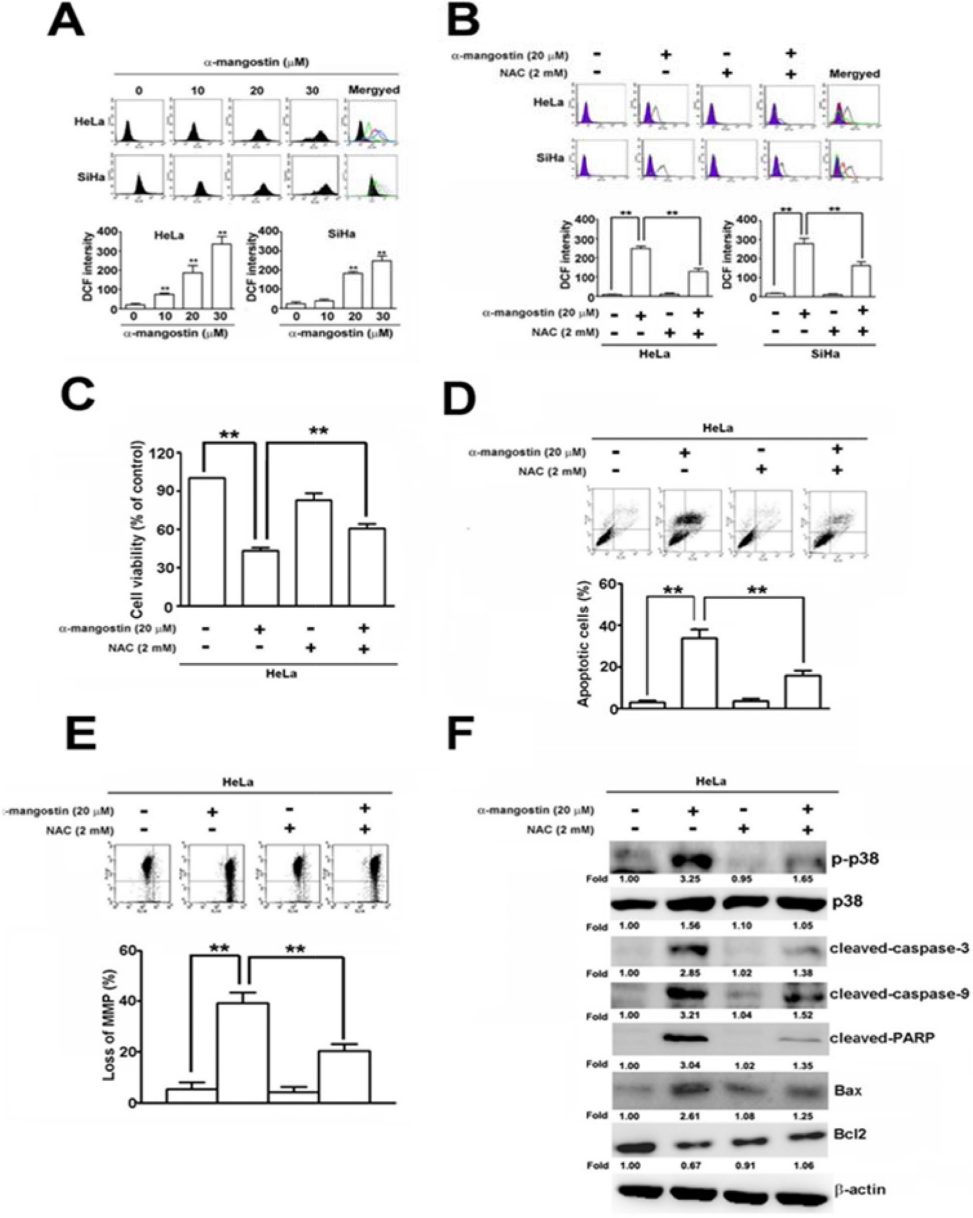
ROS are involved in α-mangostin-induced apoptotic cell death in cervical cancer cells (**A**) Cells were treated with increased concentrations of α-mangostin (0, 10, 20 and 30 μM) for 24 h. ROS content was determined according to the intensity of DCF within cells by flow cytometry. (**B**–**F**) Cells were pretreated with or without 2 mM NAC for 2 h, and then treated with or without 20 μM α-mangostin for 24 h. (B) ROS content was determined by a flow cytometry. (C) Cell viability was determined by MTT assay. (D) Apoptotic cell death was performed by Annexin V/PI staining. (E) The mitochondrial membrane potential was detected by JC-1 staining. (F) Expressions of p-p38, p38, and indicated apoptosis-related proteins were determined by immunoblotting. **P < 0.01.

Accumulated evidence has demonstrated that ROS play critical roles in stress-induced cell death by different stimuli [31], which raises a question about whether ROS regulate p38-mediated apoptosis caused by α-mangostin. ROS content was dramatically enhanced by increased concentrations of α-mangostin (Figure 4A). Addition of a ROS scavenger, N-acetyl-L-cysteine (NAC), significantly reduced α-mangostin-induced ROS in both HeLa and SiHa cells (Figure 4B). Moreover, addition of NAC also significantly suppressed α-mangostin-induced cell death (Figure 4C), apoptosis (Figure 4D), as well as loss of MMP (Figure 4E). In particular, NAC inhibited α-mangostin-induced phosphorylation of p38 and apoptotic responses, including decreased amounts of cleaved-caspase-3, cleaved-caspase-9, cleaved- PARP, Bax and increased amounts of Bcl-2 (Figure 4F). Taken together, these results demonstrate that α-mangostin enhances ROS generation, leading to activation of p38 and induction of apoptotic cell death in cervical cancer cells.

### ASK1/p38 signaling pathway is involved in α-mangostin-induced apoptotic cell death and loss of MMP

**Figure 5:**
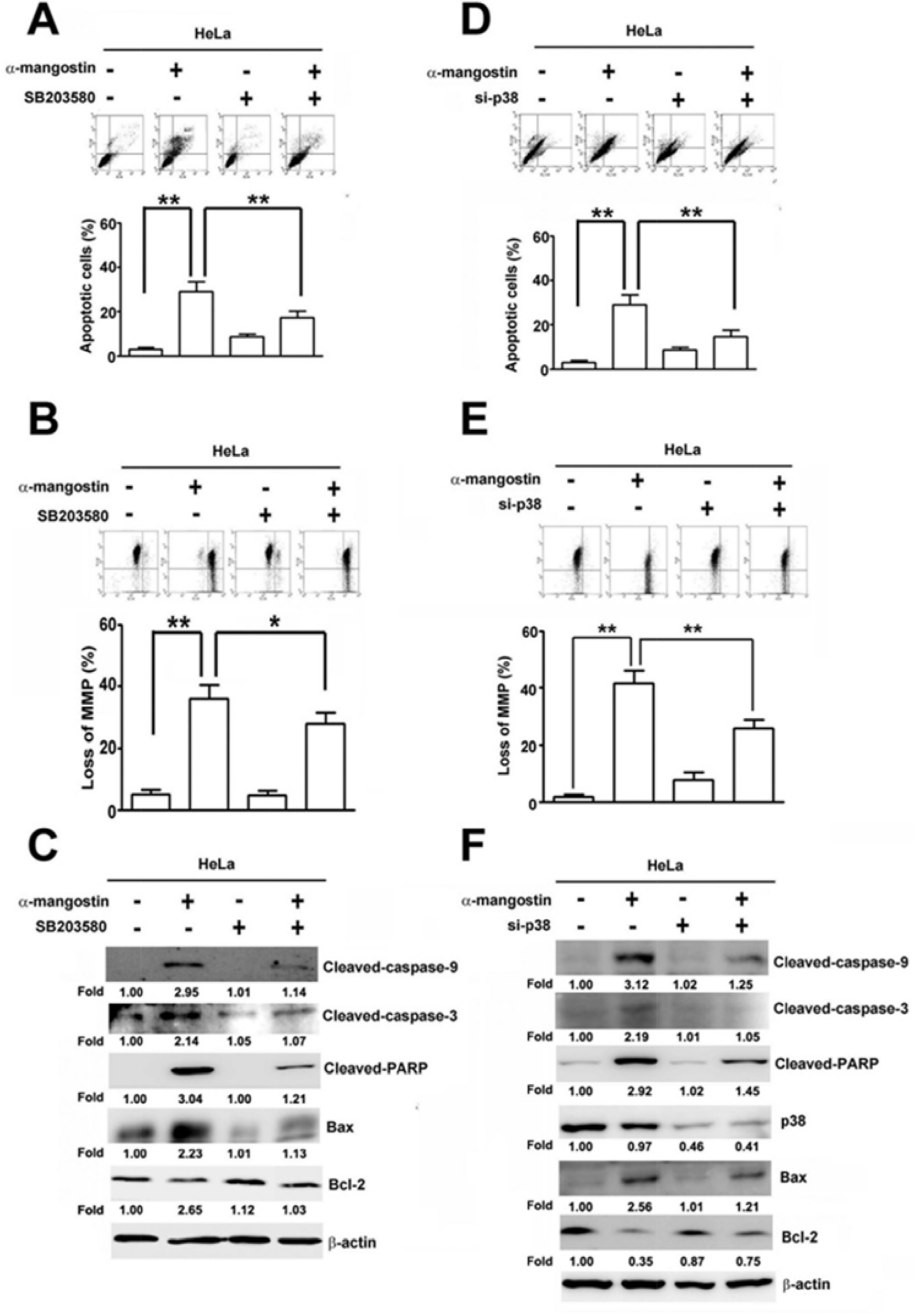
p38 is involved in α-mangostin-induced apoptotic cell death in cervical cancer cells (**A**–**C**) HeLa cells were pretreated with or without 50 μM SB203580, a p38 inhibitor, for 2 h, and then treated with or without 20 μM α-mangostin for 24 h. (**D–F**) HeLa cells were transfected with or without the specific siRNA against p38 for 24 h, and then the cells were treated with or without 20 μM α-mangostin for 24 h. After treatment, apoptotic cell death (A, D), mitochondrial membrane potential (B, E), and expressions of indicated apoptosis-related proteins (C, F) were determined. **P < 0.01.

**Figure 6:**
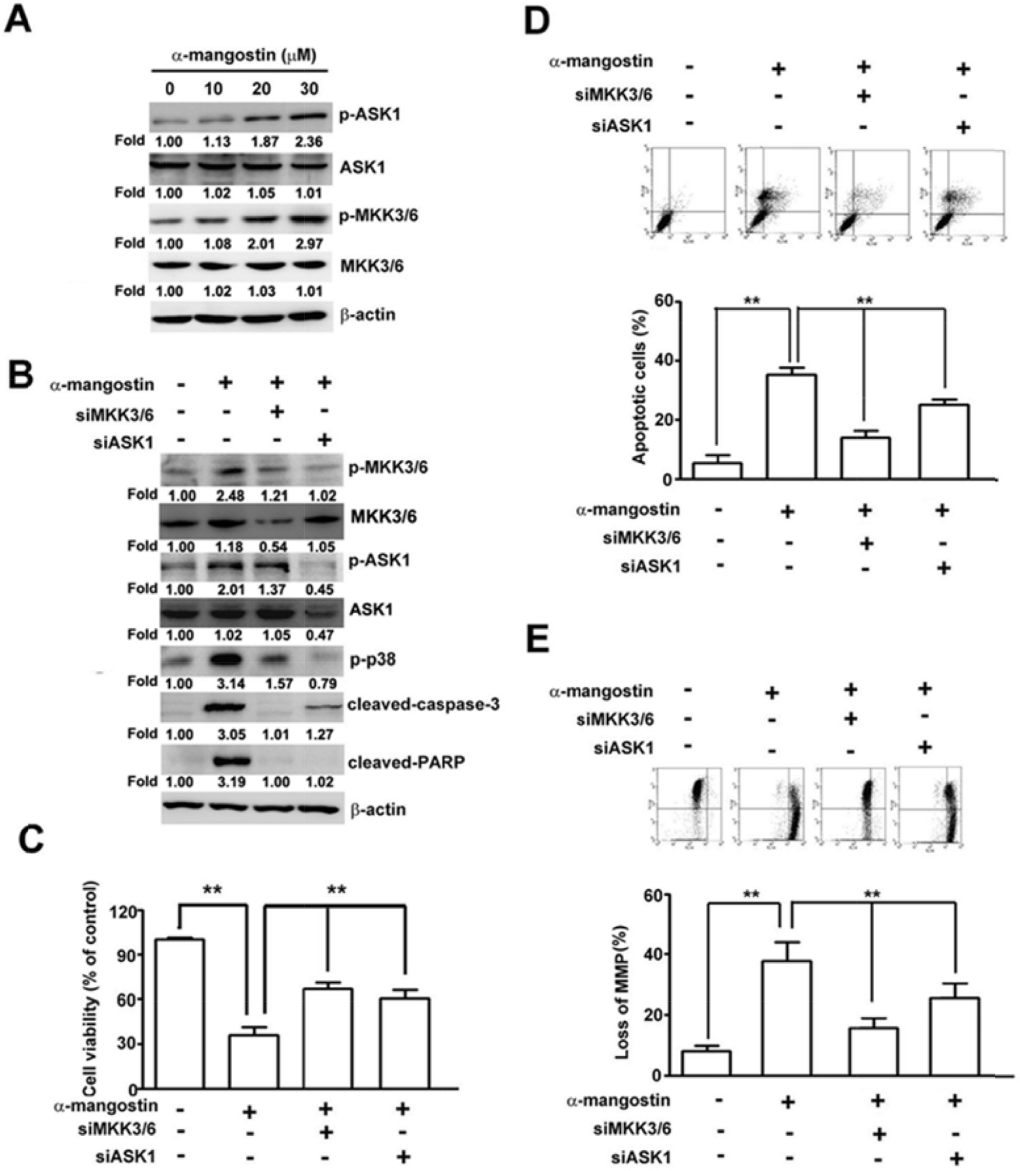
ASK1-MKK3/6-p38 cascade mediates α-mangostin-induced apoptotic cell death in cervical cancer cells (**A**) HeLa cells were treated with increased concentrations of α-mangostin (0, 10, 20 and 30 μM) for 24 h. Amounts of phosphorylated and total ASK1 and MKK3/6 were examined by immunoblotting. β-actin is shown as an internal control. (**B**–**E**) HeLa cells were transfected with or without the specific siRNAs against ASK1 or MKK3/6 for 24 h, and then the cells were treated with or without 20 μM α-mangostin for 24 h. After treatment, expressions of indicated proteins (B), cell viability (C), apoptotic cell death (D), and mitochondrial membrane potential (E) were determined. **P < 0.01.

To further clarify the roles of ASK1/p38 signaling pathway in α-mangostin-induced cell death, p38 and its upstream kinases, ASK1 and MKK3/6, were disrupted by specific inhibitors or siRNAs to determine their effects on apoptotic responses in cervical cancer cells. Disrupting p38 kinase activity by SB203580 significantly suppressed α-mangostin-induced apoptotic cell death (Figure 5A), loss of MMP (Figure 5B), the amounts of cleaved-caspase-9, cleaved-caspase-3, cleaved-PARP, and Bax, and enhanced Bcl-2 levels in HeLa cells (Figure 5C). Similarly, these α-mangostin-induced apoptotic responses were also abrogated by silencing p38 with transfection of specific siRNA against p38 (Figure 5D–5F). ASK1 and MKK3/6 are known as upstream kinases of the p38 signaling pathway [25], thus their effects on α-mangostin-induced apoptotic cell death were investigated further. Phosphorylation of both ASK1 and MKK3/6 (p-ASK1 and p-MKK3/6) were significantly increased by increased concentration of α-mangostin (Figure 6A). Moreover, knockdown of MKK3/6 or ASK1 by siRNAs resulted in decreasing α-mangostin-induced p-p38, cleaved-caspase-3, cleaved-PARP (Figure 6B), and cell death (Figure 6C). Knockdown of MKK3/6 or ASK1 significantly reduced α-mangostin-induced apoptotic cell death (Figure 6D), and loss of MMP (Figure 6E). Taken together, these results reveal that the ASK1/MKK3/6/p38 signaling cascade regulates α-mangostin-induced damage of the integrity of mitochondria and apoptotic cell death.

### Alpha-mangostin suppresses tumor growth in accordance with activation of ASK1/p38 and caspase cascades in the mouse xenograft model of cervical cancer

**Figure 7:**
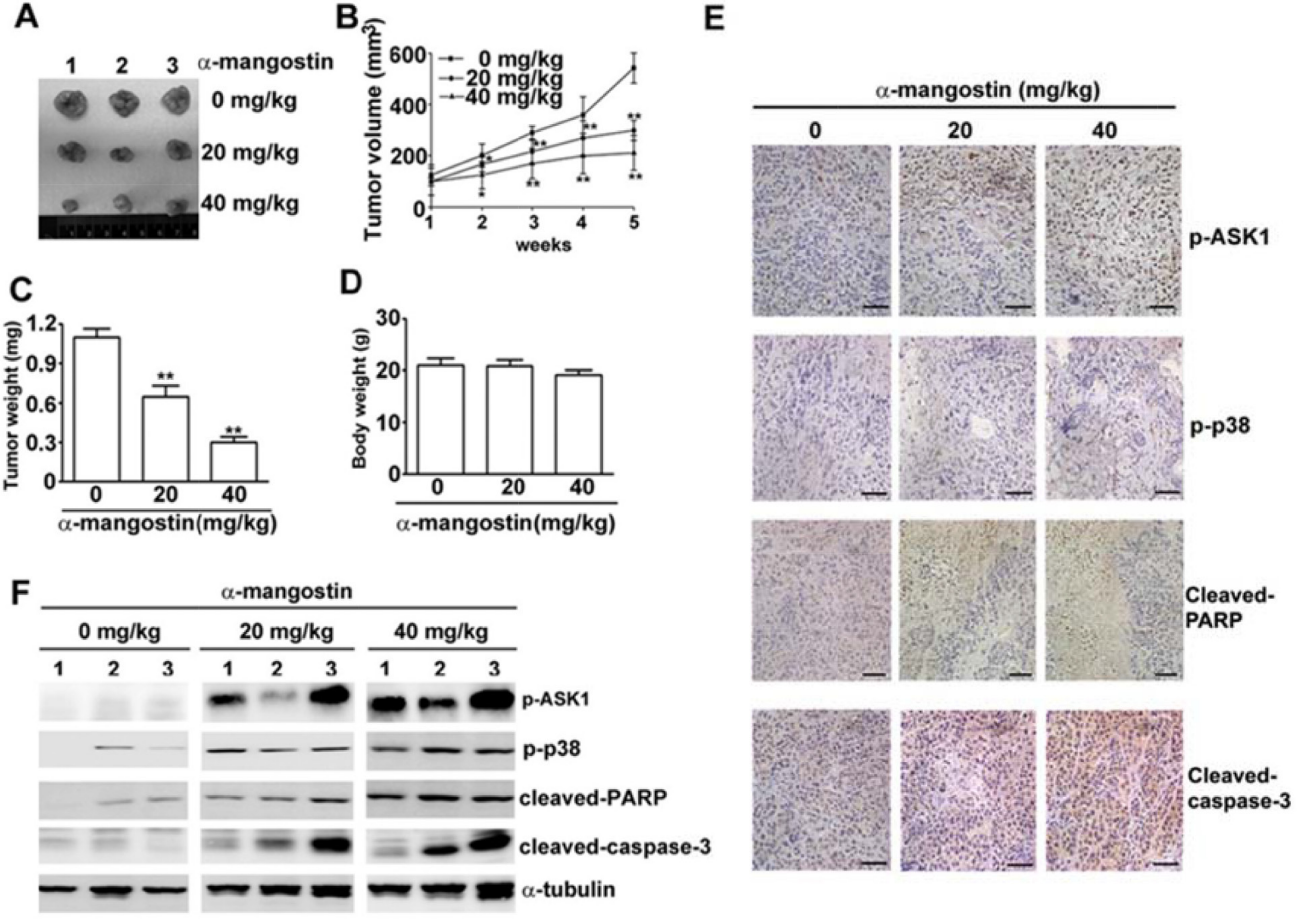
Alpha-mangostin suppresses tumor growth in accordance with activation of ASK1/p38 and caspase cascade in a xenograft model of cervical cancer Nude mice (BALB/c nu/nu) were treated with DMSO (vehicle) or α-mangostin (20 or 40 mg/kg) every week after an initial subcutaneous injection of HeLa cells. (**A**) Representative tumors isolated from mice at 5 weeks after initiation of treatment are shown. (**B**) Tumor volume at each time interval was measured during treatment. The tumor (**C**) and body (**D**) weight of each mouse were measured at the end of the treatment. Expressions of p-ASK1, p-p38, cleaved-PARP, and cleaved-caspase-3 in the tumor mass were examined by IHC staining (**E**) and by immunoblotting (**F**). Representative results are shown. **P < 0.01.

Alpha-mangostin suppresses tumor growth in accordance with activation of ASK1/p38 and caspase cascades in the mouse xenograft model of cervical cancer. A mouse xenograft model of cervical cancer was established to validate the correlation of ASK1/p38 and α-mangostin-induced apoptotic cell death *in vivo*. HeLa cervical cancer cells were inoculated into nude mice subcutaneously. Subsequent treatment of the HeLa cells-inoculated mice with 20 or 40 mg/kg of α-mangostin reduced tumor size (Figure 7A), tumor volume (Figure 7B), and tumor weight (Figure 7C) without altering mice body weight (Figure 7D). IHC staining showed that p-ASK1, p-p38, cleaved-PARP, and cleaved-caspase-3 increased in tissue sections from the inoculated tumors (Figure 7E). The protein levels of p-ASK1 and p-p38, cleaved-PARP, and cleaved-caspase-3 also increased in tumor masses (Figure 7F). These results suggest that α-mangostin represses cervical cancer growth via ASK1/p38 mediated caspase-3 activation *in vivo*.

**Figure 8:**
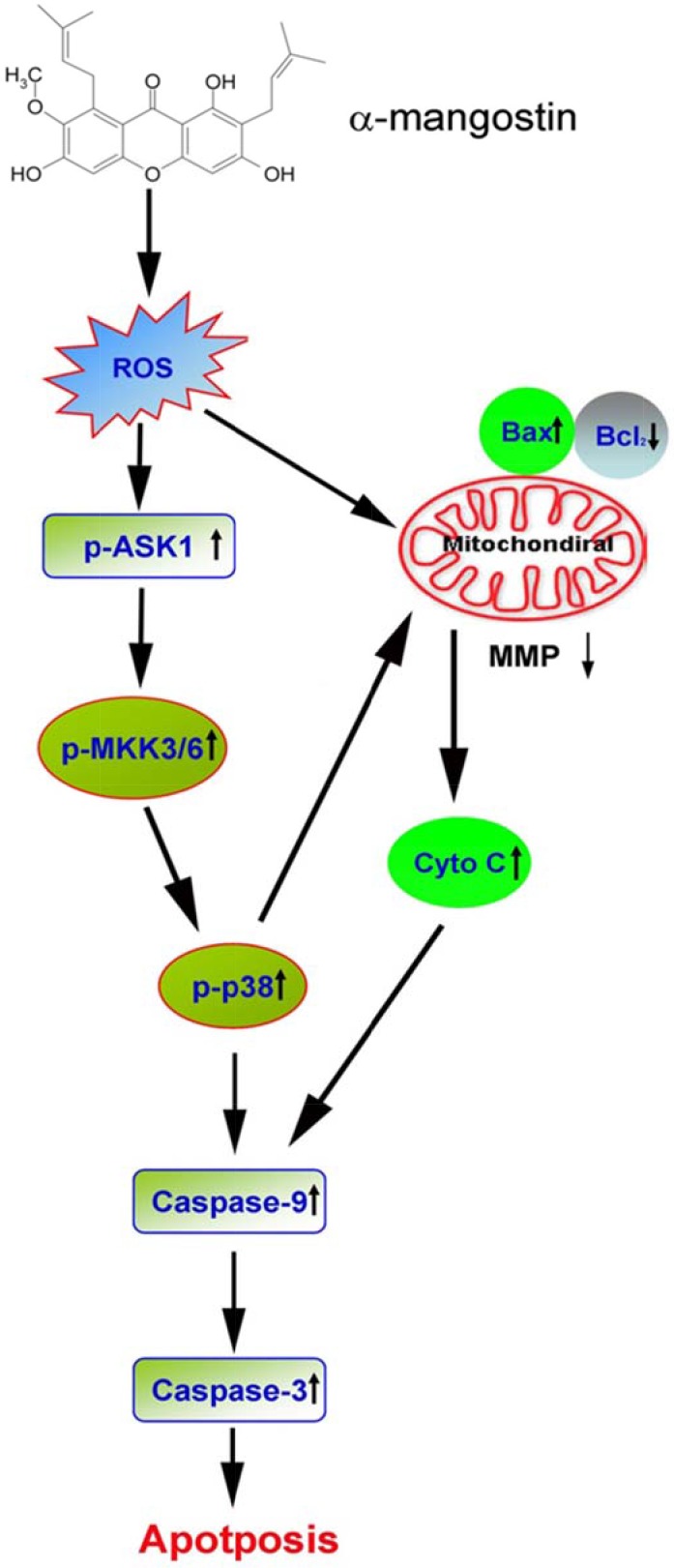
A proposed model of α-mangostin-induced apoptosis in cervical cancer The scheme illustrates the proposed mechanism of α-mangostin-induced apoptosis in cervical cancer. Alpha-mangostin enhances ROS content to activate ASK/p38 signaling pathway and damage the integrity of mitochondria, including loss of MMP, increase of Bax and cytochrome C release, and decrease of Bcl-2, leading to activation of caspase-9/caspase-3 cascade and induction of apoptosis in cervical cancer cells.

In summary, results of these experiments demonstrate that α-mangostin enhances ROS amounts to activate the ASK/p38 signaling pathway and damage the integrity of mitochondria, including loss of MMP, increase of Bax and cytochrome C release, and decrease of Bcl-2, leading to activation of caspase-9/caspase-3 cascade, and induction of apoptosis in cervical cancer cells (Figure 8).

## DISCUSSION

Results of the present study have shown that α-mangostin induces generation of ROS to activate the ASK/p38 signaling pathway and rupture mitochondria, followed by loss of MMP, increase of Bax and cytochrome C release, decrease of Bcl-2, leading to triggering caspase-9/caspase-3 cascade and inducing apoptosis in cervical cancer cells. In addition to dominant apoptotic cell death, parts of cells underwent necrotic cell death after treatment of α-mangostin (Figure 1C), that might be the probable reason why addition of a pan-caspase inhibitor, Z-VAD, did not dramatically rescue cells from α-mangostin-induced cell death (Figure 1F and Figure 1G). These results provide new insight into the molecular mechanism of α-mangostin-induced apoptosis, and reveal a potential therapeutic application of α-mangostin in the treatment of cervical cancer. Furthermore, α-mangostin has been shown to have properties of antioxidant [8], anti-inflammation [11], anti-cancer [16, 32], as well as anti-metastasis [33, 34], suggesting that dietary α-mangostin in combination with drugs treatment might potentially benefit the outcome of cancer patients.

Accumulated evidence has shown that α-mangostin effectively induces apoptotic cell death and suppresses tumor growth, invasion, and metastasis in some cancer types. Different signaling pathways are involved in the anti-cancer and anti-metastasis activities of α-mangostin. Alpha-mangostin significantly inhibits intracellular fatty acid accumulation and induces apoptosis by suppressing intracellular fatty acid synthase (FAS) expression and activity in MCF-7 and MDA-MB-231 breast cancer cells [35]. A recent study demonstrated that α-mangostin induces apoptosis through up-regulation of HER2/PI3K/Akt and p38 pathways, and down-regulation of ERK1/2 MAPK signaling pathway in T47D breast cancer cells [17]. Alpha-mangostin repressed metastasis of PC-3 prostate carcinoma cells by inhibiting matrix metalloproteinase-2/9 (MMP-2/MMP-9) and urokinase-plasminogen expression via suppression of the JNK1/2 signaling pathway and inhibition of NF-κB and AP-1 binding activity [34], Also, in another study, α-mangostin suppressed lipopolysaccharide-induced invasion by reducing MMP-2/MMP-9 expression and increasing E-cadherin expression through inhibition of the ERK signaling pathway in MIAPaCa-2 and BxPC-3 pancreatic cancer cells [13]. Additionally, down-regulation of the PI3K/Akt pathway was demonstrated to be involved in α-mangostin-reduced viability, EMT, MMP-2/MMP-9 expression, and invasion in BxPc-3 and Panc-1 pancreatic cancer cells [20].

Results of the present study demonstrate that α-mangostin induces apoptotic cell death through activation of ASK1/MKK3/6/p38 signaling pathway, and inhibition of this pathway by specific inhibitors or siRNAs significantly attenuates α-mangostin-induced apoptosis in HeLa and SiHa cervical cancer cells. Alpha-mangostin-induced apoptosis via up-regulation of p38 has been also observed in T47D breast cancer cells [17]. Interestingly, our previous study [29] showed that α-mangostin causes apoptotic cell death through inhibition of p38 MAPK pathway in SK-Hep-1 hepatocellular carcinoma cells. The roles of p38 MAPK in regulating apoptosis are divergent, and both pro-apoptotic and anti-apoptotic effects of p38 have been reported. For instance, p38 induced apoptosis in response to different cellular stresses by transcriptional up-regulation of the pro-apoptotic genes of the Bcl-2 family [36]. In addition, it has been reported that p38-mediated phosphorylation and activation of Bax induced its mitochondrial translocation in cardiomyocytes upon simulated ischemia [37] and in HepG2 hepatocellular carcinoma cells treated with different pro-apoptotic stimuli [38]. The expressions of Fas and Fas ligand (FasL), which contribute to induction of apoptosis, were also mediated by p38 in response to different T-cell stimuli [39] and in AML-12 hepatocytes expressing hepatitis B virus X protein [40]. In contrast, p38 was shown to have anti-apoptotic effects in response to different stimuli. Up-regulation of anti-apoptotic members of the Bcl-2 family, including Bcl-2 and Bcl-xL, has been reported to be mediated by p38 in carbon monoxide-protected ischemia/reperfusion injury of the lung [41], and in HaCaT keratinocytes exposed to ultraviolet A (UVA) [42]. In addition, p38 also induces tumor dormancy, resulting in a survival state and a quiescent state related to drug resistance through activation of the p38α-ATF6α-Rheb-mTOR pathway [43]. Furthermore, p38 was shown to participate in cell-cycle arrest and facilitate DNA repair at the G2/M checkpoint, which may antagonize chemotherapy-induced DNA damage, leading to apoptosis resistance in cancer cells [44]. Taken together, the contributions of p38 to both pro-apoptotic and anti-apoptotic events through these different mechanisms may be dependent upon different stimuli and cell types.

ROS have been shown to trigger activation of p38, which negatively regulates the malignant transformation induced by oncogenic H-Ras. Since p38 serves as a sensor of ROS, it is likely to be important in the inhibition of tumor initiation [45]. Alpha-mangostin has been known to possess the antioxidant and antitumor property, probably via reduction of NF-κB, Stat3, and MMP9 expression and inhibits pancreatic tumor growth *in vivo* [14]. For example, α-mangostin inhibits hypoxia-driven ROS-induced aggression against pancreatic cancer cells by reducing ROS production, which leads to inhibition of HIF-1α stabilization, GLI1 expression, and EMT [13]. Alpha-mangostin has been shown to reduce ROS production and suppress VEGF-induced permeability, proliferation, migration tube formation, and angiogenesis in bovine retinal endothelial cells (REC) [46]. In contrast, in the present study, α-mangostin enhanced ROS content in a dose-dependent manner and activated p38 to induce the mitochondria-mediated apoptotic cell death of cervical cancer cells. Alpha-mangostin-induced ROS generation has also been observed to trigger mitochondria-mediated apoptotic cell death, including increased cytochrome c release and activation of caspase-9/caspase-3 cascade in MDA-MB-231 breast cancer cells [47]. Similarly, γ-mangostin, also a xanthonoid from the mangosteen tree, enhances intracellular ROS production and induces apoptosis in HT29 colorectal adenocarcinoma cells [48]. It has been reported that ROS increases activity of p38 MAPK and expression of connexin 43 (Cx43) in cardiomyocytes. However, knockdown of p38 and Cx43 results in a significant decrease in ROS production. These results suggest that there is a positive-feedback loop between ROS and p38 MAPK-Cx43 for the sustained activation of p38 MAPK, leading to loss of cell division in cardiomyocytes [49]. Taken together, α-mangostin not only protects cells from stress or toxin-induced cell death by scavenging ROS, but also induces ROS production to trigger apoptotic cell death, which may most likely be attributable to different stimuli and cell types.

The present study has revealed that α-mangostin enhances ROS production and results in activation of the ASK/p38 signaling pathway and mitochondria-dependent caspase-9/caspase-3 cascade, leading to induction of apoptotic cell death in cervical cancer cells (Figure 8). We also showed that administration of α-mangostin inhibits tumor growth in accordance with increased levels of p-ASK1, p-p38, cleaved-PARP, and cleaved-caspase-3 in a mouse xenograft model of cervical cancer. These results unveil the molecular mechanisms of α-mangostin-induced apoptotic cell death and provide new insight into the potential therapeutic application of α-mangostin in cervical cancer.

## MATERIALS AND METHODS

### Reagents and antibodies

Alpha-mangostin (99% purity), dimethyl sulfoxide (DMSO), 3-(4,5-dimethylthiazol-2-yl)-2,5-diphenyltetrazolium bromide (MTT), N-acetyl-L-cysteine (NAC), and 5,5′,6,6′-Tetrachloro-1,1′,3,3′-tetraethylbenzimidazol-carbocyanine iodide (JC-1), were purchased from Sigma (St. Louis, MO, USA). Z-VAD, a pan-caspase inhibitor, was obtained from BioVision (Mountain View, CA, USA). The kinase inhibitors, PD98059 to MEK, SB203580 to p38, and SP600125 to JNK, were bought from Calbiochem (San Diego, CA). The antibodies against phosphory-ERK, phosphory-p38, phosphory-JNK, ERK, p38, JNK, phosphory-MKK3/6, MKK3/6, Bcl-2, Bax, cytochrome C, COX4, α-tubulin, β-actin, siRNA-ERK, siRNA-JNK and siRNA-p38, siRNA-ASK1, siRNA-MKK3/6 were purchased from Santa Cruz Biotechnology (Dallas, Texas). The antibodies against cleaved-caspase-3, cleaved-caspase-9, cleaved-poly-ADP-ribose polymerase (PARP), phosphory-ASK1 and ASK1 were purchased from Cell Signaling (Danvers, MA, USA). Horseradish peroxidase-labeled anti-mouse and anti-rabbit secondary antibodies were bought from Promega (Madison, WI, USA).

### Cell culture

Human cervical cancer cell lines, SiHa (ATCC HTB35) was obtained from the American Type Culture Collection (ATCC; Manassas, VA, USA) and HeLa (BCRC No 60005) was obtained from the Bioresources Collection and Research Center, Food Industry Research and Development Institute (Hsinchu, Taiwan), these cells were cultured in Dulbecco's Modified Eagle Medium (DMEM) supplemented with 10% fetal bovine serum (FBS; HyClone, Logan, UT, USA), 2 mM glutamine, 100 U/ml penicillin and 100 μg/ml streptomycin (Sigma), at 37°C in a humidified atmosphere with 5% CO_2_. Cells were passaged every 2-3 days to maintain exponential growth.

### Cell viability assay

Cell viability was determined by MTT assay [50]. Cells were seeded at a density of 2 × 10^4^ cells/well in a 24-well plate on the day before the experiment. The cells were treated with various concentrations of α-mangostin for 24 or 48 h. Subsequently, the medium was replaced with fresh medium containing MTT (0.5 mg/ml) for 4 h. The number of viable cells was proportional to the amounts of formazan, a reduction of MTT, by dehydrogenases in the mitochondria within live cells. The produced formazan was dissolved in isopropanol and measured at 570 nm by a Multiskan MS ELSA reader (LabSystems, Helsinki, Finland). The relative cell number was normalized by absorbance from the untreated cells.

### Annexin V/propidium iodide (PI) apoptosis assay

Apoptosis was detected using an FITC-labeled Annexin V/PI Apoptosis Detection kit (BD Biosciences, CA, USA) according to the manufacturer's instruction manual [51]. In brief, cells were stained with FITC-labeled Annexin V and PI and then analyzed with a flow cytometer (FACSCalibur, BD Biosciences). The percentage of apoptosis was calculated by Annexin V-positive cells, including early apoptotic (Annexin V-positive, PI-negative) and late apoptotic (Annexin V-positive, PI- positive) cells.

### siRNA transefection

HeLa cells were seeded in 6-cm plates at a density of 4 × 10^5^ cells/well. The siRNA-ERK (200 nM), siRNA-p38 (200 nM), siRNA-JNK (200 nM), siRNA-MKK3/6 (200 nM), siRNA-ASK1 (200 nM) were introduced plates into the cells using Turbofect reagents (Fermentas, Carlsbad, CA) according to the manufacturer's protocol. Cells were transfected for 48 h before treatment with α-mangostin and then collected for further experiments.

### Western blotting

The cell lysate was prepared by lysing the cells in RIPA buffer (50 mM Tris at pH 7.5, 150 mM NaCl, 1 mM EDTA, 0.25% Na-deoxycholate, 1% NP-40, 1 mM NaF, 1 mM Na_3_VO_4_, 1 mM PMSF, 1 μg/ml aprotinin) by sonication. The soluble extraction was collected from the supernatant after centrifugation at 15000 g for 10 minutes. Equal amounts of protein extracts were separated by 10 or 12.5% SDS-PAGE and transferred onto a polyvinylidene fluoride (PVDF) membrane (Millipore, Belford, MA, USA). After blocking, the membrane was hybridized with the antibodies against cleaved-caspase-3 (1:1000), cleaved-caspase-9 (1:1000), cleaved-PARP (1:1000), phosphory-ERK (1:1000), phosphory-p38 (1:1000), phosphory-JNK (1:1000), ERK (1:2000), p38 (1:1000), JNK (1:1000), phosphory-ASK1 (1:1000), ASK1 (1:1000), phosphory-MKK3/6 (1:1000), MKK3/6 (1:1000), Bcl-2 (1:1000), Bax (1:1000), cytochrome C (1:500), COX4 (1:1000), α-tubulin (1:2000) and β-actin (1:2000). The reaction was visualized using ECL (Pierce) and detected using a Luminescent Image Analyzer LAS-4000 mini.

### Mitochondrial membrane potential (MMP) assay

MMP was detected with JC-1, a dual-emission mitochondrial dye. JC-1 accumulates in mitochondria depending on the potential indicated by a fluorescence emission shift from green (JC-1 monomer) to red (JC-1 aggregate). Consequently, mitochondrial depolarization (loss of MMP) is indicated by a decrease in the intensity of red fluorescence. Cells were incubated with 10 μg/ml JC-1 dye at 37°C for 30 minutes and then washed with phosphate-buffered saline (PBS) for 5 minutes. Subsequently, cells were analyzed with a FACSCalibur flow cytometer (BD Biosciences).

### Reactive oxygen species (ROS) assay

ROS content was determined using the 2′,7′-dichlorofluorescin diacetate (DCFDA) After permeating into the cells, DCFDA was deacetylated by cellular esterases to a non-fluorescent compound, which was later oxidized by ROS into a fluorescent 2′, 7′-dichlorofluorescein (DCF). In brief, cells were incubated with 20 μM DCFDA at 37°C for 30 min. After washing with PBS, cells were analyzed using a FACSCalibur flow cytometer (BD Biosciences) and ROS were indicated with the intensity of DCF.

### Immunohistochemical (IHC) staining

Tissue sections were deparaffinized, rehydrated, and quenched using endogenous tissue peroxides. Subsequently, the tissue slides were incubated with 1% hydrogen peroxide at room temperature for 10 minutes, and then blocked with 5% bovine serum albumin (BSA) for 30 minutes. The tissue slides were hybridized with primary antibody (1:100) at room temperature for 1 hour, followed by hybridization with HRP-conjugated secondary antibody (1:1000) for 30 minutes. The tissue slides were then visualized by addition of peroxidase substrate for 1–10 minutes until the desired stain intensity developed. Subsequently, the tissue sections were counterstained with hematoxylin. The images were observed and photographed under microscopy

### *In vivo* xenograft animal model

The protocols of animal experiments were approved by the Institutional Animal Care and Use Committee of Chung Shan Medical University, and the animals were cared for in accordance with institutional guidelines. Female nude mice (BALB/c nu/nu), approximately 5-weeks old, were obtained from the National Laboratory Animal Center (Taipei, Taiwan). HeLa cells (5 × 10^6^/100 μl) were washed suspended in PBS, and inoculated subcutaneously into the left flank of the mice (*n* = 5). After 7 days, the HeLa cells-inoculated mice (*n* = 5) were randomized into three groups, in which they were intraperitoneally (i.p.) injected with 0, 20, or 40 mg/kg of α-mangostin (3 times/week). The control group received an equal amount of DMSO as the experimental groups. Tumor size and body weight were measured after at 7-day intervals. Tumor volume was calculated with the formula 0.5236 × L (W)^2^, where L and W represent the long and short axes of the tumor, respectively. The mice were sacrificed and the tumors were excised, weighed, photographed, and sectioned for further experiments.

### Statistical analysis

The results are presented as mean ± standard error (S.E.) from three independent experiments. Data were analyzed using the student's *t*-test. *P*-value < 0.05 was considered to be statistically significant.
